# Parental care, loss of paternity and circulating levels of testosterone and corticosterone in a socially monogamous song bird

**DOI:** 10.1186/1742-9994-11-11

**Published:** 2014-02-12

**Authors:** Camila P Villavicencio, Beate Apfelbeck, Wolfgang Goymann

**Affiliations:** 1 Abteilung für Verhaltensneurobiologie, Max-Planck-Institut für Ornithologie, Eberhard-Gwinner-Str. 6a, D-82319 Seewiesen, Germany; 2 Current address: Institute of Biodiversity, Animal Health and Comparative Medicine, University of Glasgow, G12 8QQ Glasgow, UK

**Keywords:** Steroid hormones, Black redstart, Aves, Mate-guarding, Extra-pair paternity

## Abstract

**Introduction:**

In biparental birds testosterone levels of males are typically high during the mating phase and decrease during the parental phase. Testosterone implants may enhance mating behaviors, increase the likelihood of males to engage in extra-pair mating behavior and may reduce paternal care. Thus, sex steroids such as testosterone influence reproductive behaviors. Little is known, however, as to whether the more subtle differences in physiological concentrations of testosterone that occur between individuals are related to differences in paternal care, extra-pair behavior, and genetic paternity between those males. Here, we investigate these relationships in the male black redstart (*Phoenicurus ochruros*), a socially monogamous songbird with a low breeding synchrony. We used nestling provisioning as a proxy for parental care behavior and genetic paternity loss as a proxy for the efficiency of mate-guarding.

**Results:**

There was no relationship between nestling provisioning and paternity loss of males. Baseline and gonadotropin releasing hormone (GnRH)-induced levels of testosterone, but not baseline corticosterone, were significantly higher during the mating than during the provisioning phase. Males fed more often when temperatures decreased and fed less when they sang more, but we found no correlation between parental behavior and baseline or GnRH-induced testosterone, and baseline corticosterone – both measured during either the mating or the parental phase. However, males that experienced loss of paternity had lower levels of testosterone during the provisioning phase than males that did not lose paternity. Further, males that lost paternity also expressed higher baseline levels of corticosterone.

**Conclusions:**

Physiological differences in testosterone or baseline corticosterone were not related to differences in parental care, suggesting that the variation of testosterone within a physiological range may not relate to the degree of paternal care in this species. However, the profile of both hormones may indicate quality traits that influence the likelihood of the respective male to lose paternity.

## Introduction

Steroid hormones regulate traits central to growth, metabolism and reproduction, and thus may influence a wide range of life-history traits (e.g. [1]). For instance, in many seasonally reproducing species testosterone levels are high at the beginning of the breeding season, thus potentially enhancing mating and aggressive behaviors [2-5]. The majority of bird species are socially monogamous and biparental, i.e. males and females form pairs and both partners provision their offspring [6]. However, during the last two decades research using genetic tools revealed that females and males frequently engage in extra-pair behavior leading to extra-pair fertilization [7]. Therefore, males taking care of their young may diminish their possibilities to gain extra-pair paternity, in particular in species that breed asynchronously. Because sex steroids influence reproductive behaviors, an adequate seasonal modulation of testosterone can be important for optimal breeding performance [8] for several reasons. First, high testosterone levels during the parental phase can interfere with paternal behavior [9]; in particular, experimentally elevated levels of testosterone have been shown to reduce paternal care [10-19]. Second, high levels of testosterone during the mating season may increase the likelihood of males to show extra-pair behavior [1,20,21]. This has been corroborated by a comparative study indicating that males of bird species with higher rates of extra-pair paternity expressed higher peak levels of testosterone [22]. Third, testosterone may enhance copulatory behavior [23] and mate guarding, in which males attempt to prevent their female partners from engaging in extra-pair fertilizations [10,24].

Hormones other than testosterone have also been suggested to play a role in the regulation of reproductive traits. For example, levels of baseline corticosterone (a hormone involved in energy metabolism and the stress response [25]) have been shown to relate to clutch mass, number of nestlings and their growth rate [26], or to parental behavior and reproductive success [27-31]. However the nature of this relationship can change across stage or context [29,32] rendering it difficult to come up with clear predictions.

Although experimental studies have established that there is an apparent close association between steroid hormones and reproductive behaviors, little is known about the natural variation of reproductive traits in relation to physiological variation of hormone levels [33,34]. Testosterone manipulation studies often work with supraphysiological doses [35] and do not take into account the huge variation of testosterone levels among males. To better understand the role of hormones in the variation of life history traits, it is thus necessary to focus on the individual variation of hormones within their natural range [36]. So far, only few studies have investigated the individual variation between testosterone (or corticosterone) levels and parental behavior [36]. In addition to baseline concentrations of plasma hormones, another – so far little explored – hormonal parameter that can be used to assess the link with individual variation of traits are gonadotropin releasing hormone (GnRH) induced levels of testosterone, which – depending on the testicular status – can be an indicator of maximum production of testosterone [37-40]. In dark-eyed juncos (*Junco hyemalis*), the increase of testosterone after a GnRH challenge correlated positively with aggressive behavior, and negatively with parental care, suggesting that natural variation in testosterone (and not only pharmacological manipulations) can mediate the trade-off between mating and parental effort in this species [8]. The junco study implies that the potential of an individual to increase testosterone release (e.g. the individual variation in GnRH-induced increase of testosterone) could account for individual variation and may be more relevant than baseline levels of testosterone in modulating mating and parental behaviors.

Studies that combine measures of testosterone, parental care and genetic paternity within the same population are still rare [41], but could be important in clarifying the role of this sex steroid in the mediation of traits related to paternal care and extra-pair behavior [1]. Here, we assess the relationship between natural variation in baseline and GnRH-induced levels of testosterone, and baseline corticosterone with parental care and extra-pair paternity in male black redstarts (*Phoenicurus ochruros*), which are socially monogamous songbirds. From the male’s point of view extra-pair behavior has two main aspects: first, gaining additional offspring with one or more extra-pair females and second, mate guarding during which a male prevents his female partner to engage in extra-pair behavior with other males. Because we could not monitor the whole population of black redstarts we could not reliably assess paternity gain. We thus focused on paternity loss as a result of ineffective mate guarding. Therefore, we investigated whether natural variation in levels of testosterone and corticosterone were related to paternal care and paternity loss. Opportunities for extra-pair fertilizations are typically higher when the parental period overlaps with the opportunity of males to gain additional mates [7], i.e. when breeding attempts of females are not synchronized and when females have more than one brood per breeding season. Female black redstarts are not synchronous with regard to successfully initiating a first clutch and they can raise up to three broods per season with clutch size ranging from 2 to 6 eggs [42,43]. In our population, the initiation of first clutches range from end of April until beginning of June, probably depending on female condition, quality of the territory, nest site and weather conditions (C. Villavicencio, W. Goymann and B. Apfelbeck, unpublished observations). The rate of extra-pair paternity in black redstarts has not yet been quantified, but this is essential to assess a possible relationship between mating and parental behavior and testosterone. Previous studies in this species have indicated that testosterone levels show large variation among males [44], but this variation was not related to territorial aggression [45,46] and thus could be related to mating behavior and/or paternal care [47].

In the current study, we first aimed to assess the rate of extra-pair paternity in black redstarts. Second, we investigated whether paternal care was related to baseline or GnRH-induced levels of testosterone and baseline concentrations of corticosterone measured during the mating and parental periods. In addition, we assessed other parameters affecting parental care such as singing behavior, which is mainly related to territory defense and mate attraction [48]. Third, we asked whether paternal care was related to loss of paternity to assess whether the level of care could be a response to certainty of genetic paternity. Finally, we asked whether baseline and GnRH-induced levels of testosterone or baseline corticosterone concentrations measured during the mating and parental periods were related to the loss of paternity in this species.

## Results

### Extra-pair paternity

The paternity analyses revealed that 30.2% of nests (N = 16 out of 53) in the study population contained extra-pair offspring. Overall, 28.8% of the nestlings (N = 64 out of 222) were not sired by the social father. Taking into account all nests, the proportion of extra-pair offspring in one nest was 27.1 ± 11.4% (mean ± 95% confidence interval).

### Paternal care

**Figure 1:**
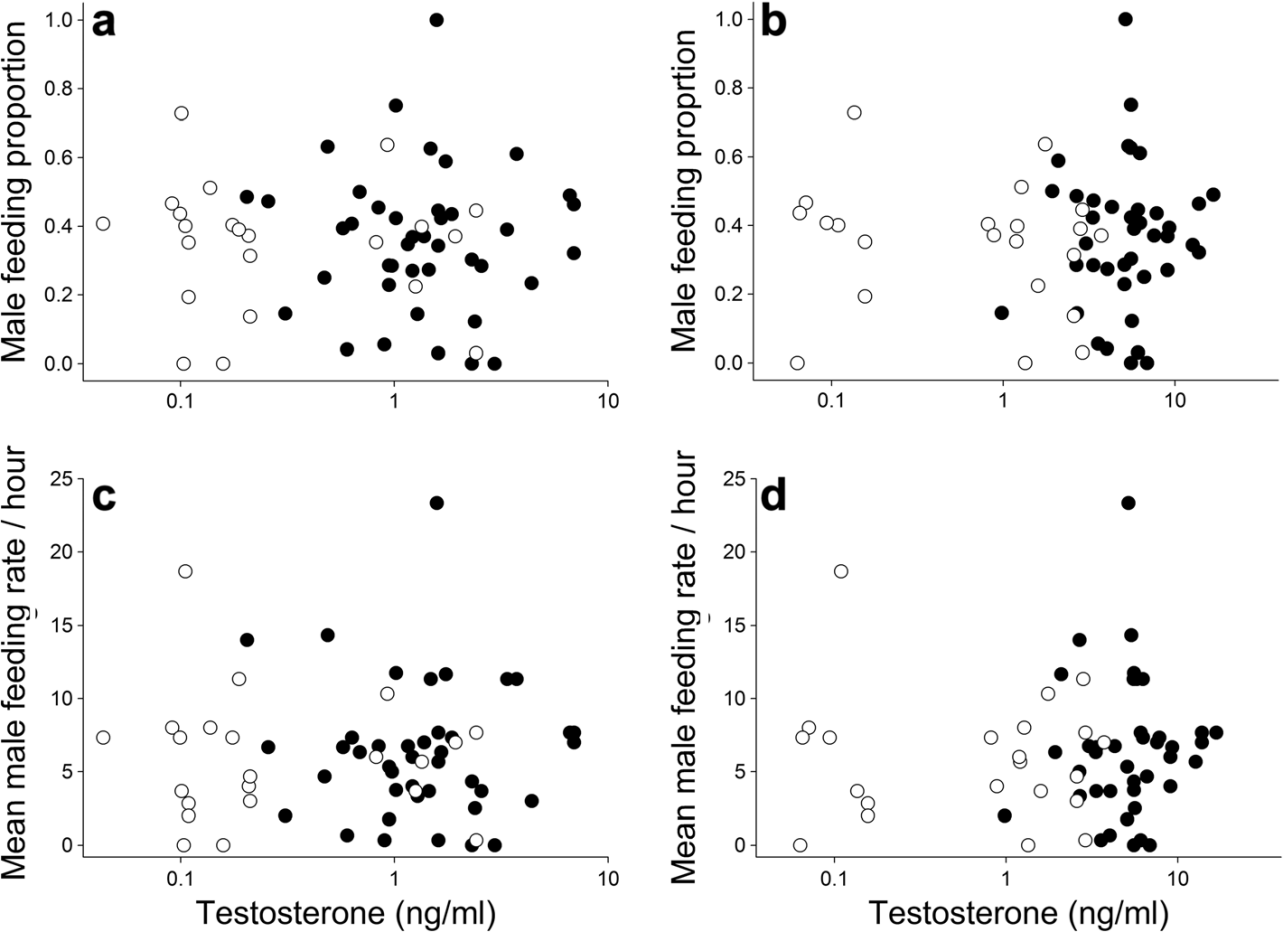
**Parental care and testosterone.** Relative and absolute male parental care was not related to **(a,c)** post capture testosterone and **(b,d)** GnRH-induced testosterone concentrations (log scale). Black dots represent males sampled during mating; open dots represent males sampled when they were feeding nestlings.

The feeding rates of females and males were not significantly correlated (linear mixed model: χ^2^ = 1.85, P = 0.174; Pearson’s correlation, r = −0.03, P = 0.83). We first investigated which factors were related to the relative degree of paternal care, i.e. the proportion of male parental care: The mean effect size of post-capture testosterone on paternal behavior was close to zero and also the credible interval for the effect size included zero (Table 1). Thus, based on a Bayesian framework, post-capture levels of testosterone were not related to paternal behavior (Table 1, Figure 1). Also GnRH-induced testosterone concentrations were not predictive for paternal behavior, as the effect size was very low and the credible interval included zero (Table 2, Figure 1). The only factors that were related to the relative degree of paternal care, in both models, were ambient temperature and song frequency: the lower the ambient temperature, the higher the degree of paternal care, and the more a male sang the less it contributed to parental care (Tables 1 and 2). When we used the absolute feeding rate of males rather than the proportion only song frequency had a significant negative impact on paternal care (Tables 1 and 2).

**Table 1:** Male nestling provisioning in relation to post-capture testosterone, breeding and environmental parameters

	**Relative male provisioning rate**	**Absolute male provisioning rate**
	**Estimate [Credible intervals (2.5% – 97.5%)]**	**Estimate [Credible intervals (2.5% – 97.5%)]**
Intercept (Stage feeding)	0.08 [−0.83 – 1.05]	9.14 [3.08 – 15.7]
Stage mating	0.43 [−0.05 – 0.9]	0.69 [−2.14 – 3.42]
Post-capture testosterone	0.0001 [−0.00016 – 0.0004]	0.0003 [−0.002 – 0.002]
Brood	0.15 [−0.21 – 0.51]	−0.19 [−2.14 – 3.42]
Rain	−0.002 [−0.008 – 0.005]	−0.02 [−0.06 – 0.04]
Temperature	**−0.054 [−0.096 – −0.01]**	−0.17 [−0.47 – 0.1]
Cloud cover	0.003 [−0.003 – 0.008]	0.003 [−0.04 – 0.04]
**Songs**	**−0.05 [−0.08 – −0.03]**	**−0.14 [−0.23 – −0.06]**
Testosterone: stage	−0.0002 [−0.0006 – 0.0001]	−0.0002 [−0.002 – 0.002]

The left column shows the relative male provisioning rate (proportion) and the right column the absolute male provisioning rate, each with the corresponding Bayesian estimate and its’ credible intervals. Estimates of cofactors refer to differences from the intercept estimate, which represents the feeding stage. If 0 (zero) is not included in the credible intervals there is an effect of this parameter on the dependent variable. ‘Significant’ effects with credible intervals not including zero are shown in bold.

**Table 2:** Male nestling provisioning in relation to GnRH-induced testosterone, breeding and environmental parameters

	**Relative male provisioning rate**	**Absolute male provisioning rate**
	**Estimate [Credible intervals (2.5% – 97.5%)]**	**Estimate [Credible intervals (2.5% – 97.5%)]**
Intercept (Stage feeding)	−0.51 [−1.96 – 0.93]	9.17 [2.31 – 15.71]
Stage	−0.07 [−4.99 – 4.98]	0.89 [−2.63 – 4.37]
GnRH-induced testosterone	0.11 [−0.06 – 0.28]	0.0003 [−0.001 – 0.002]
Brood	0.19 [−0.19 – 0.57]	0.08 [−2.16 – 2.4]
Rain	−0.002 [−0.009 – 0.004]	−0.01 [−0.06 – 0.04]
Temperature	**−0.06 [−0.1 – −0.02]**	−0.21 [−0.49 – 0.09]
Clouds cover	0.003 [−0.003 – 0.008]	0.002 [−0.04 – 0.04]
**Songs**	**−0.06 [−0.08 – −0.03]**	**−0.14 [−0.23 – −0.05]**
Testosterone: stage	0.00004 [−0.59 – 0.58]	−0.0003 [−0.002 – 0.001]

The left column shows the relative male provisioning rate (proportion) and the right column the absolute male provisioning rate, each with the corresponding Bayesian estimate and its’ credible intervals. Estimates of cofactors refer to differences from the intercept estimate, which represents the feeding stage. If 0 (zero) is not included in the credible intervals there is an effect of this parameter on the dependent variable. ‘Significant’ effects with credible intervals not including zero are shown in bold.

**Figure 2:**
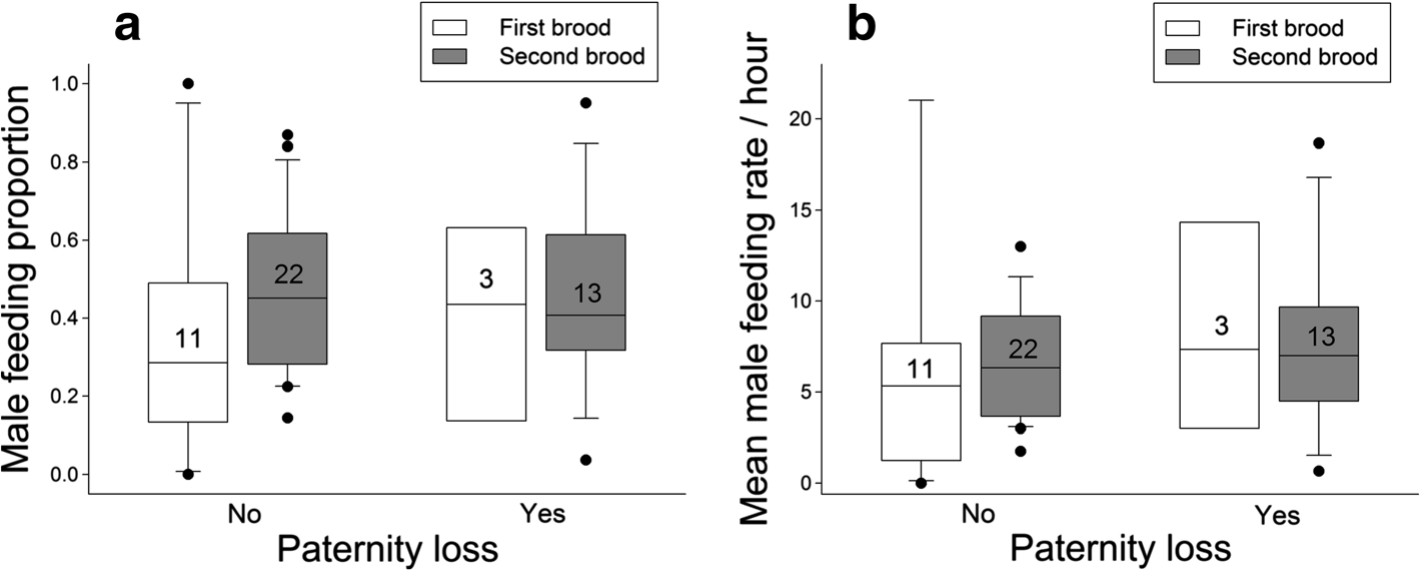
**Parental care and paternity loss.** Medians, 10th, 25th, 75th, 90th percentiles, and outliers of **(a)** relative and **(b)** absolute male parental care in relation to paternity loss. There was no difference of paternal care behavior regardless of whether a male lost paternity or not, neither in the first (open box) nor in the second brood (grey box).

In the next analysis we included all birds for which we had data on the relatedness between the social father and nestlings to investigate if the degree of paternal care was related to paternity loss. Because ambient temperature and song seemed to influence paternal care (see above) we included these two variables in this follow-up model. There was no effect of paternity loss on relative paternal care, neither in first nor in second broods (Table 3, Figure 2). Similar to the previous models, ambient temperature and song frequency were negatively related to relative paternal care, while the proportion of male care increased with the number of nestlings (Table 3). When investigating absolute male feeding rates the results were similar: males fed less when they sang more and they fed more depending on the number and age of nestlings (Table 3).

**Table 3:** Male nestling provisioning in relation to paternity loss, environmental parameters and song behavior

	**Relative male provisioning rate**	**Absolute male provisioning rate**
	**Estimate [Credible intervals (2.5% – 97.5%)]**	**Estimate [Credible intervals (2.5% – 97.5%)]**
Intercept (No paternity loss)	−1.45 [−3.06 – 0.17]	−2.78 [−11.49 – 6.27]
Paternity loss	0.22 [−0.51 – 0.98]	0.07 [−3.14 – 3.25]
Temperature	**−0.06 [−0.1 – −0.01]**	−0.16 [−0.48 – 0.15]
Brood	−0.07 [−0.45 – 0.32]	−1.28 [−3.68 – 1.16]
Age of nestlings	0.05 [−0.01 – 0.11]	**0.68 [0.28 – 1.09]**
**Number of nestlings**	**0.37 [0.1 – 0.65]**	**1.66 [0.38 – 2.97]**
**Songs**	**−0.05 [−0.07 – −0.02]**	**−0.11 [−0.21 – −0.02]**

The left column shows the relative male provisioning rate (proportion) and the right column the absolute male provisioning rate, each with the corresponding Bayesian estimate and its’ credible intervals. Estimates of cofactors refer to differences from the intercept estimate, which represents no paternity loss. If 0 (zero) is not included within the credible intervals there is a ‘significant’ effect of this parameter on the dependent variable. ‘Significant’ effects with credible intervals not including zero are shown in bold.

### Testosterone and paternity loss

**Figure 3:**
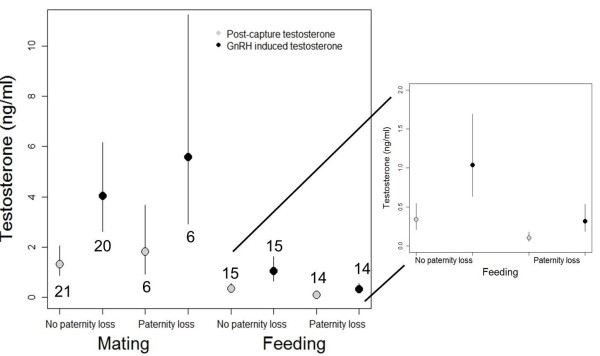
**Testosterone and paternity loss.** Back transformed means (± 95% credible intervals) of post-capture and GnRH-induced testosterone levels did not differ between males that lost or did not lose paternity, when sampled during mating. When sampled during the nestling provisioning phase males that lost paternity had lower baseline and GnRH-induced levels of testosterone than males that did not lose paternity (inset). Testosterone levels increased after the GnRH-challenge in all groups. Grey dots represent post-capture testosterone levels; black dots represent GnRH-induced testosterone levels.

In a third step, we were interested whether paternity loss as a measure of the degree of mate-guarding efficiency was related to testosterone concentrations. The Bayesian effect sizes and credible intervals indicated that testosterone concentrations were higher after a GnRH-challenge (Table 4, Figure 3) and higher during the mating stage than during the parenting stage (Table 4, Figure 3). Furthermore, the effect sizes and credible intervals for the interaction between paternity loss and breeding stage indicated that, during the nestling provisioning phase, males that had experienced a loss in genetic paternity expressed lower levels of testosterone than males that did not lose paternity (Figure 3 inset). This was not the case during the mating stage. There also was an overall effect of paternity loss on testosterone concentrations, but this was mainly driven by the lower levels of testosterone in males that lost genetic paternity during the parental stage. Brood sequence was not related to testosterone concentrations (Table 4).

**Table 4:** Testosterone concentrations in relation to paternity loss

	**Estimate**	**Credible intervals (2.5%)**	**Credible intervals (97.5%)**
Intercept (no GnRH injection)	6.15	5.42	6.85
**GnRH injection**	0.9	**0.56**	**1.25**
**Paternity loss**	−1.08	**−1.79**	**−0.38**
**Stage**	1.11	**0.5**	**1.73**
Brood	−0.08	−0.58	0.44
Male age	−0.36	−0.99	0.27
**Paternity loss: stage**	1.45	**0.6**	**2.29**
GnRH injection: stage	0.46	−0.06	0.96

The second column shows the estimates which indicate the direction of the relationship. The third and fourth columns shows the credible intervals (Bayesian), if zero is not included within the credible intervals there is a ‘significant’ effect of this parameter on the dependent variable. Estimates of cofactors refer to differences from the intercept estimate, which represents no GnRH injection. ‘Significant’ differences are shown in bold.

### Corticosterone, parental care and paternity loss

**Figure 4:**
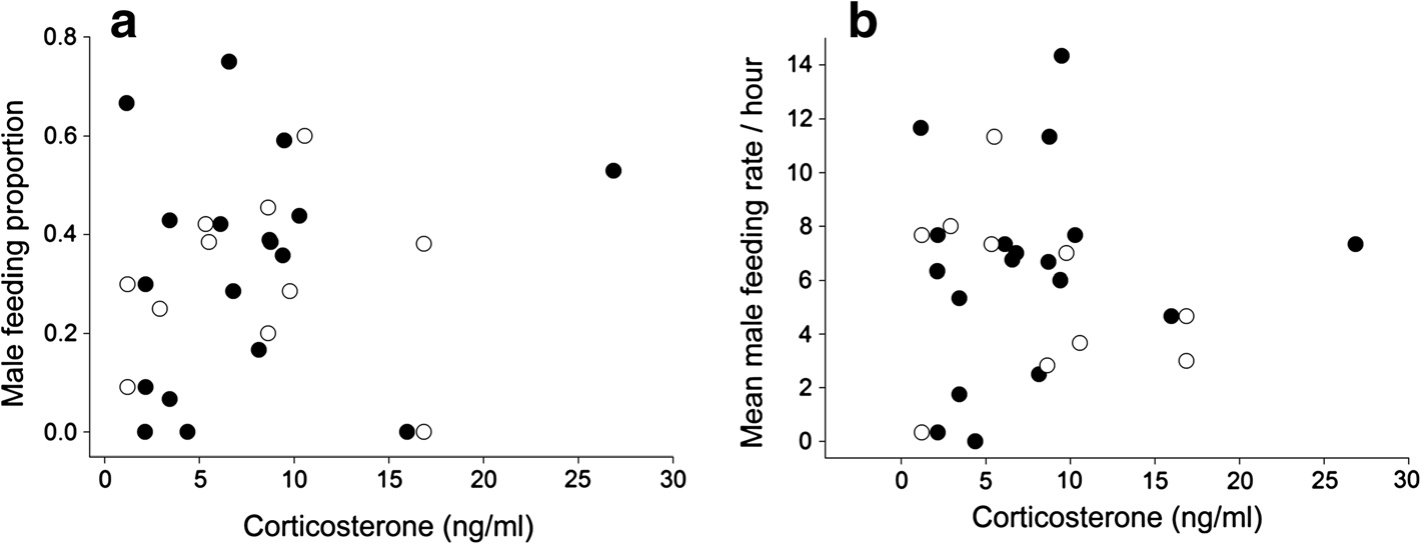
**Parental care and corticosterone. (a)** Relative and **(b)** absolute male parental care was not related to plasma levels of corticosterone. Black dots represent males sampled during mating; open dots represent males sampled when they were feeding nestlings.

**Figure 5:**
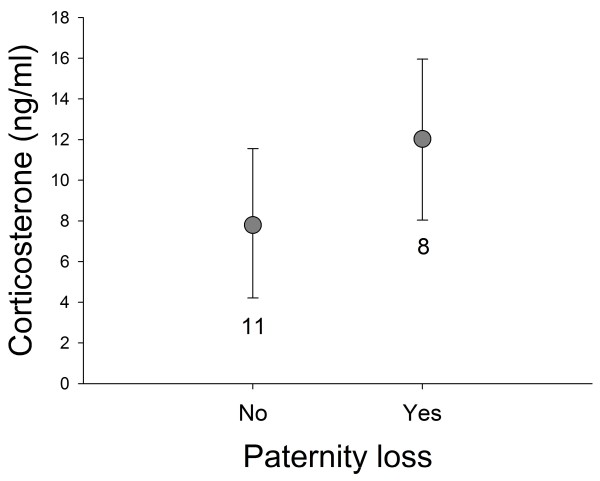
**Corticosterone and paternity loss.** Back transformed means (± 95% credible intervals) of corticosterone were significantly higher in males that lost paternity than in males that did not lose paternity.

Finally, we assessed factors that were related to baseline corticosterone concentrations. Corticosterone levels of males did not differ between breeding stages (Table 5, Figure 4), relative degree of male parental care (Table 5, Figure 4) or brood sequence. When investigating absolute male nestling provisioning rates, the results were the same: no relationship between corticosterone and male nestling provisioning rates (see Table 5). There was also no relation between corticosterone and testosterone concentrations, a relationship that was tested separately (Table 6). However, from the follow-up model where we included the subset of all birds for which we had data on the genetic relatedness between the social father and the nestlings to investigate if corticosterone was related to paternity loss, we inferred that males that had lost genetic paternity expressed significantly higher levels of corticosterone than males that did not lose paternity (Table 6, Figure 5).

**Table 5:** Corticosterone levels in relation to the relative proportion of male nestling provisioning

	**Relative male provisioning rate**	**Absolute male provisioning rate**
	**Estimate [Credible intervals (2.5% – 97.5%)]**	**Estimate [Credible intervals (2.5% – 97.5%)]**
Intercept (Stage feeding)	8.53 [7.96 – 9.12]	8.69 [8.03 – 9.35]
Stage	0.07 [−0.28 – 0.41]	0.1 [−0.29 – 0.46]
Male provisioning rate	0.93 [−0.5 – 2.33]	0.01 [−0.07 – 0.08]
Brood	−0.22 [−0.75 – 0.28]	−0.04 [−0.48 – 0.41]

The left column shows the relative male provisioning rate (proportion) and the right column the absolute male provisioning rate, each with the corresponding Bayesian estimate and its’ credible intervals. Estimates of cofactors refer to differences from the intercept estimate, which represents the feeding stage. If zero is not included in the credible intervals there is a ‘significant’ effect of this parameter on the dependent variable. We found no 'significant' effects.

**Table 6:** Corticosterone levels in relation to testosterone and paternity loss

	**Estimate**	**Credible intervals (2.5%)**	**Credible intervals (97.5%)**
Intercept	8.84	8.41	9.26
Testosterone	−0.00005	−0.0002	0.00009
Intercept (no paternity loss)	7796	3986	11727
**Paternity loss**	4238	**200**	**8130**

They were assessed in separate model and the intercept is shown for each model. The second column shows the estimates which indicate the direction of the relationship. The third and fourth columns shows the credible intervals (Bayesian), if zero is not included in the credible intervals there is a ‘significant’ effect of this parameter on the dependent variable, which are shown in bold.

## Discussion

The results of this study suggest that male black redstarts face a relatively high degree of genetic paternity loss within socially monogamous birds [7,49]. The relative degree of paternal care in redstarts increased with lower ambient temperature and decreased with song output. Also the absolute feeding rate was negatively related to song output, but did not vary with ambient temperature. Neither the relative nor the absolute male feeding rate varied with any of the physiological measures, i.e. post-capture or GnRH-induced levels of testosterone, or baseline corticosterone. Also, relatedness with the nestlings did not influence the provisioning behavior of social fathers. However, males that lost paternity had lower levels of testosterone during the parental phase and had overall higher levels of corticosterone than males that did not lose paternity. These data suggest that the hormonal state of males may be related to mate guarding and describe the susceptibility of males to extra-pair behavior of the female.

### Parental care, testosterone and corticosterone

Our data suggest that the natural variation in testosterone concentrations does not influence male provisioning behavior of black redstarts, because the degree of parental care and testosterone – including the potential to produce testosterone (GnRH-induced levels) did not covary (see Figure 1). A suppressive effect of high levels of testosterone on paternal care has been found in testosterone manipulation studies. However, it is not clear whether the effects on paternal care may have been caused by pharmacological levels of the hormone, which may be apparent especially few days after the implantation [19,35]. To date, only few studies have related the natural physiological variance in testosterone concentrations to paternal care: similar to our study, paternal behavior was not related to post-capture testosterone in European starlings [50], barn swallows [41], northern cardinals [51] and dark eyed juncos [8]. Similar to northern cardinals [51], but unlike dark eyed-juncos [8] the GnRH-induced increase in testosterone was also not related to paternal care in black redstarts. The absence of a relationship between parental care and GnRH-induced testosterone levels in black redstarts and northern cardinals on the one hand, and the presence of such a relationship in dark eyed-juncos on the other hand, is difficult to compare: In northern cardinals and in our study male and female behavior was analyzed together and feeding behavior was assessed during three consecutive days; in contrast, in the dark-eyed junco study the female was removed prior to the observation during which only males provisioned the nestlings. It is unlikely that lack of statistical power prevented us from detecting a biologically meaningful correlation between testosterone and paternal care. First, our sample size for testosterone during the provisioning phase was similar to the dark-eyed junco study (dark-eved juncos: N = 25; black redstarts: N = 21) and during the mating phase our sample consisted of 41 birds. Second, an *a priori* power analysis for a simple correlation aiming for a medium effect size (r = 0.5) with a power of 0.80 requires a sample of 29 birds. Thus, for the mating stage our analysis had a power of approximately 0.92 and for the parental phase a power of 0.64, rendering it rather unlikely that we may have missed a medium effect of testosterone on parental behavior. Because we sampled birds during two breeding sub-stages (mating and provisioning stage) this leads to two conclusions. First, testosterone concentrations during the mating phase did not predict parental care behavior in black redstarts. In *Peromyscus* mice mating-induced levels of testosterone correlated with paternal care behavior [52]. It is unknown, whether mating induces an increase in testosterone in male black redstarts. But if so, the injection of GnRH should have a similar effect on males’ testosterone concentrations. Because baseline and GnRH-induced levels of testosterone during the mating phase did not predict paternal care we consider it unlikely that testosterone is predictive of paternal care in this species. Second, the actual levels of testosterone expressed during the nestling provisioning phase were also not indicative of the degree of paternal care. Furthermore, even though testosterone levels were higher when black redstarts fed nestlings during the first brood than when they were feeding the second brood [45,53], males did not contribute differently to parental care between first and second broods (Figure 2). Although there is the possibility that males may differ with respect to the amount of food they provided during each nest visit (we could not quantify this for a sufficient number of birds), our data suggest that the natural variation in testosterone levels does not have a large impact on parental behavior in this species. Thus, it is questionable whether testosterone is involved in mediating a trade-off between mating and paternal behavior in black redstarts. Definitely, more studies on different species are needed to resolve the question whether physiological concentrations of testosterone are related to paternal care. In particular, we need to explore the relationship between the reaction norm of an individual’s testosterone concentration and its potential relationship to parental behavior.

Also baseline corticosterone concentrations did not vary with paternal behavior, which is in contrast to some other studies in which baseline corticosterone predicted fitness and feeding behavior [27,29-31]. Because corticosterone is a metabolic hormone one may expect higher levels to be associated with a higher feeding frequency [30]. However, in black redstarts this was not the case. Corticosterone concentrations can be very dynamic and the relationship of corticosterone and parental care can change across stages [29,32]. In an effort to account for stage-related differences we measured corticosterone levels during two breeding stages. However, corticosterone levels did not differ between stages and did not correlate with parental behavior.

Interestingly, environmental factors rather than physiological parameters influenced male feeding behavior, as the relative proportion of the male feeding was higher at lower ambient temperatures. Previous studies have reported an effect of ambient temperature on parental care [54-57], but unlike our study, they did not find differences in the relative provisioning rate of females and males. In addition, males contributed proportionally more if there were more young in the nest, unlike for example hen harriers (*Circus cyaneus*) where female but not male provisioning rate varies positively with brood size [58]. However, provisioning rates typically increase with brood size (e.g. [54,55,59,60]). Also, feeding behavior was negatively related to singing behavior, i.e. males fed more when they sang less (like, for example in the reed bunting, *Emberiza schoeniclus*; [48]). Because a male cannot sing and feed nestlings at the same time it is not surprising that males that spent more time singing contributed less to offspring care.

### Paternity loss and parental care

Loss of paternity was relatively high (~30% of nests) in black redstarts compared to other birds species with a similar mating system [7,49]. Such high levels of paternity loss may result from a low degree of breeding synchrony and multiple-broodedness [7,49] in this species. However, paternal care did not differ between males that lost paternity and those that did not, suggesting that males did not adjust the degree of paternal care to paternity. This finding is in line with the observation that male birds in general do not discriminate between their own and extra-pair young [61].

### Testosterone, corticosterone and paternity loss

Males that lost paternity expressed lower concentrations of testosterone than males that did not lose paternity when they were feeding nestlings (mainly of the second brood, see Figure 5). This suggests a possible link between testosterone and paternity loss, which may be related to the effectiveness of mate-guarding. Possibly, testosterone concentrations during the late breeding season indicate some trait related to male condition, whereby males with lower condition start to shut-down their reproductive system earlier than males in better condition. This explanation is supported by the data on GnRH-induced levels of testosterone during the nesting stage: males that lost paternity were not able to increase testosterone as much as males that did not lose paternity. Thus, the regression of the reproductive system of males that lost paternity was further advanced than that of males that did not lose paternity. Also the corticosterone data are consistent with this idea: males with higher levels of baseline corticosterone (possibly indicating a higher allostatic load *sensu*[62] experienced a higher degree of paternity loss (see also [63] and [29] for similar results). Alternatively, males that showed a higher investment in mating behavior maintained higher levels of testosterone for a longer period of time, and hence, were more capable of preventing their female from engaging in extra-pair fertilizations. Thus, the seasonal dynamics of an individuals’ testosterone profile or individual hormonal reaction norms may be more important in relation to fitness-relevant traits than currently recognized. So far, the rate at which testosterone concentrations decline during the breeding season within individuals remains largely unknown. On a population level, the decline in testosterone has been reported to correlate with the duration of mating behaviors and the breeding season [64]. Surprisingly, there are only few other published studies that relate testosterone concentrations and extra-pair behavior (see also [1] for further discussion of this topic). Possibly, there is a publication bias, i.e. non-significant relationships between testosterone and paternity may not be published (e.g. [65] did not report testosterone because there was no relationship of testosterone with extra-pair paternity). To date, we are only aware of one study relating natural variation in testosterone levels and extra-pair paternity [66]. They found no correlation between testosterone levels and paternity loss in barn swallows. Another study measured cuckoldry risk or extra-pair opportunities in Seychelle warblers (*Acrocephalus sechellensis*) and did not find a relationship with testosterone [67]. Three studies have analyzed the effect of testosterone implants on extra-pair behavior. In two studies of dark-eyed juncos testosterone implants led to an increase in extra-pair paternity [21,68]. In contrast, testosterone implants reduced extra-pair paternity in blue tits (*Cyanistes caeruleus*[69]), which might have been due to negative feedback of testosterone leading to a shut-down of sperm production.

## Conclusions

In summary, the data on black redstarts provide little evidence that the level of care could be a response to certainty of paternity. Importantly, there is also no indication that high levels of testosterone would interfere with parental care. Nevertheless, the maintenance of high levels of testosterone and low levels of corticosterone throughout the breeding season may be related to effective mate-guarding or good condition in male black redstarts, thus reducing the likelihood of losing paternity. Further, unlike in dark-eyed juncos [8] a GnRH-induced increase in testosterone did not appear to explain parental care behavior. However, both baseline and GnRH-induced testosterone levels during the provisioning phase seemed to relate to paternity loss. These data suggest that in order to find relationships of hormones with fitness-relevant traits we may need to better understand the seasonal dynamics and the hormonal reaction norms of individuals. Our data also add further support to the idea that testosterone in black redstarts (and possibly other birds that are territorial during most of the year) is mainly related to mating behavior, while it does not play a major role in territorial behavior ([53,70]). Studies relating parental care, paternity and testosterone (or other hormones) within the same individuals are still scarce, but are urgently needed to better understand individual variation in life-history traits and their physiological basis.

## Material and methods

This study was conducted in a migratory population of black redstarts in Upper Bavaria, Germany, in villages in the vicinity of the Max-Planck-Institut für Ornithologie (47°N, 11°E, 500–600 m above sea level). Black redstarts of this population arrive on their breeding grounds from late March to the beginning of April. In the years in which we conducted the experiments, the first broods were raised from May 10^th^ until June 8^th^ in 2010, and from May 5^th^ until June 15^th^ in 2011. Second broods were raised between June 15^th^ until July 29^th^ in 2010 and from June 20^th^ until July 30^th^ in 2011. Black redstarts typically build their nests in houses or barns in human settlements. Females incubate the clutch of 2–6 eggs, but both parents feed nestlings and fledglings [42]. Black redstarts show delayed plumage maturation with second-year males (i.e. males in their first breeding season) resembling females and attaining their black coloration with white wing patches only after their first postnuptial molt [43].

### Field procedures and blood sampling

Black redstarts were caught in mealworm-baited traps by first luring them to the traps with a short playback of black redstart song or by placing a stuffed decoy into their territory and playing back black redstart song for 20 minutes (simulated territorial intrusion; STI). Previous studies have demonstrated that black redstarts do not increase testosterone concentrations following single or repeated simulated territorial intrusions [45,46]. Therefore, we did not expect differences in testosterone concentrations depending on the catching method. Males were caught during the mating period between April 15^th^ until June 10^th^ (n = 35) and during the parental care period between May 25^th^ until July 31^st^ (n = 34) in 2010 and 2011, 8 of these males were sampled during both stages. Three males were caught while feeding the first brood (25^th^ of May until June 11^th^), and 31 males were sampled during the second brood (June 23^rd^ until July 31^st^). A subset of males (n = 25) was sampled also for plasma levels of corticosterone, but only in 2011: for corticosterone 17 males were caught during mating and 10 males were caught while feeding their nestlings, four of these males were sampled twice. The breeding stage of males (mating or parental) was determined using behavioral and nest observations. All males caught during April were assumed to be in the mating stage, because we did not find any nests in this period. The mating stage was further confirmed *a posteriori*, after finding and back-dating the nests of the respective pairs. Males were considered in the parental stage when they had an active nest in their territory and made frequent nest visits with food in their beaks.

Immediately after catching, a blood sample from the wing vein was obtained to determine baseline corticosterone (2.4 ± 0.2 min after capture; mean ± 95% CI) and post-capture testosterone concentrations (4.6 ± 0.4 min after capture). Following the first blood sample, 50 μl of chicken GnRH-I (Bachem H 3106; 1.25 μg dissolved in 50 μl isotonic saline) was injected into the *pectoralis major* muscle for the determination of GnRH-induced testosterone levels (see also [39]). After the injection, each bird was kept in a holding bag for 30 minutes until the second blood sample was taken. Each bird was measured (body mass, right tarsus, wing and tail lengths, and width and height of the cloacal protuberance) and banded with a unique numbered aluminum ring (Vogelwarte Radolfzell) and unique color bands for individual identification. Blood samples were immediately centrifuged with a Compur Minicentrifuge (Bayer Diagnostics) to separate the plasma from blood cells. Plasma volume was measured using a Hamilton syringe and stored in 500 μl ethanol [71]. The blood cells were dissolved in Queen’s lysis buffer [72] for genetic paternity analyses and stored at room temperature. After returning from the field, plasma samples in ethanol were stored at −80°C. All experimental procedures were approved by the animal ethics committees of Upper Bavaria.

### Parental behavior

Nestling provisioning rates were used as a proxy for parental behavior. Feeding behavior of both parents was measured by direct observation of the nest. The number of nest visits of both parents was counted during one hour on three consecutive days from days 6–13 after hatching. Song frequency was also assessed during that time. Although we attempted to measure the amount of food carried by the parents, we were not able to measure this for all the individuals and for each nest visit. However, we found quite some variability within individuals regarding the amount of food they carried even within just one observation (ranging from 1 to 4 beak sizes). All observations took place in the morning from 06:00 to 11:00 hours. We only conducted observations if at least one parent was color-banded. When one parent was unbanded we assumed that all feedings visits of unbanded birds were performed by the same individual. Black redstarts typically raise two (sometimes three) broods per season, with the first nestling period lasting between May 5^th^ until June 18^th^, and the second between June 21^st^ until July 30^th^ 2010 and 2011. We observed parents during the nestling phase of 22 first and 41 second broods (see Table 7). Most nests were hard to access (or inaccessible) and hence regular nest inspections could not be done. The age of the nestlings was thus estimated by the fledging date, which is typically 14 days after hatching [43]. In addition, we collected weather data to assess if environmental parameters influence the feeding behavior of the parents. We used ambient temperature, cloud cover, and rainfall. The environmental weather parameters were obtained from the local weather center (Wetterstation Wielenbach) as means of the morning from 7:00–13:00, on the exact days we performed the nest observations.

**Table 7:** Details of the feeding protocols

**Observation dates**	**Year**	**Brood no.**	**No. of nests observed**
19-May/29-May	2010	1^st^	8
15-Jun/29-Jul	2010	2^nd^	35
11-May/9-Jun	2011	1^st^	13
15-Jun/28-Jul	2011	2^nd^	18

Date, year and number of first and second broods observed to determine parental nestling provisioning rate.

### Paternity

From the nestlings we obtained a small (ca. 5 μl) blood sample by puncturing the wing vein when they were at least one week old. In total, we collected DNA samples from 138 males, 68 females and 222 nestlings from 53 broods during the two consecutive years. For paternity analysis we used 13 microsatellite markers, combined in 4 mixes (Asμ15-ZEST, CcaTgu3, CcaTgu15, CcaTgu21, DkiB102-ZEST, TguEST09-005, TguEST09-021 [73], TG01-124, TG02-088 [74], ADCYAP1 [75], Tgu7 [76], Mcyμ4 [77], Gf06 [78], see Additional file 1: Table S1). DNA extraction was performed with NucleoSpin Blood QuickPure Kit (Machery-NagelGmbH & Co. KG, Düren, Germany). Multiplex PCR-reaction were performed with the Qiagen Multiplex PCR kit (Qiagen, Hilden, Germany) and primer mixes containing three to five primer pairs (mix A – D, Additional file 1: Table S1) at primer mix specific temperatures (52-57°C, Additional file 1: Table S1). Forward primers were labeled at their 5′ end with fluorescent dyes. Differences in amplification efficiency and dye strength of the primers were accommodated by adapting the primer concentrations in these mixes (details given in Additional file 1: Table S1). Each 10 μl multiplex PCR contained 20 – 100 ng DNA, 5 μl of the 2× Qiagen Multiplex PCR Master Mix, 1 μl of one of a primer mix and 3 μl of ddH_2_O. Cycling conditions were: 15 min initial denaturation at 95°C, 24–25 cycles (see Additional file 1: Table S1) of 30 s denaturation at 94°C, 90 s annealing at 52-57°C, and 1 min extension at 72°C, followed by 30 min completing final extension at 60°C. 1.5 μl of the PCR product was mixed with formamide containing the GeneScan 500 LIZ Size Standard and heat denatured. Fluorescently labeled PCR products were sized on a 3130 xl Genetic Analyser (Applied Biosystems, Darmstadt, Germany). Subsequently allele lengths were determined using GeneMapper 4.0 software. The most likely set of parents were searched from the pool of available males and females with CERVUS version 3.0.3 (©Field Genetics Ltd). If either the social male or social female was not sampled, the set of alleles from the nestlings and the available parent was used to infer the genetic father/mother. Because males and females were observed provisioning the nestlings, it was possible to recognize the social parents. Nestlings that had more than two mismatches with the social father were assigned to be extra-pair young, none of the nestlings had mismatches with the mother. The number of mismatched loci among extra pair nestlings was 6.4 ± 2.1 (mean ± sd). Only one mismatch loci with the social father (n = 2, after repeated genotyping) was assumed to be due to mutation [79].

### Hormone analysis

Testosterone and corticosterone concentrations were determined by radioimmunoassay following the procedures described in [71]. Samples were assayed in duplicate and distributed randomly between two assays. The extraction recovery for testosterone was 88.0% ± 5.5% (mean ± sd). Hormone concentrations were calculated with Immunofit 3.0 (Beckmann Inc., Fullerton, CA, USA). The lower detection limits of the testosterone assays were 0.35 pg/ml and 0.45 pg/ml, respectively, and all samples were above the detection limit. The intra-assay coefficients of variation were 8.7% and 13%, respectively; the intra-extraction coefficients of variation of a chicken plasma pool were 0.02% and 5.6%, respectively. The inter-assay coefficient of variation between the two assays was 14.8% and the inter-extraction coefficient of variation between the two assays was 11.1%. For corticosterone, extraction recovery was 83% ± 6% (mean ± sd). Samples were measured in duplicate in one assay. The lower detection limit of the assay was 4.78 pg/ml and all samples were above the detection limit. The intra-assay coefficient of variation was 4.1%; the intra-extraction coefficient of variation of the chicken plasma pool was 2.5%.

### Data analysis

Data were analyzed using the R (2.13.0; R Development Core Team) package “arm” [80]. To determine the relationship between parental care and testosterone, two models were used: one including post-capture testosterone and another one using GnRH-induced testosterone as independent variables. Paternal care, expressed as the proportional feeding rate of the male (male feeding rate/ total feeding rate), was the dependent variable. We used the proportion to reduce the influence of other factors (i.e. chick age). In addition, we calculated the same model using the absolute male feeding rate. Independent variables included post-capture testosterone (or log transformed GnRH-induced testosterone concentrations for the second model), breeding stage during sampling (mating or feeding) and its interaction with testosterone, brood sequence (1^st^ or 2^nd^ brood), rain, temperature, cloud cover and song frequency using a generalized linear mixed model (glmer) with a binomial distribution. We did not find any age-related difference throughout the analysis, and thus did not include male age as a factor in these models. Because we measured the feeding rate on 3 consecutive days, bird ID was included as a random effect to account for repeated measures. For the inferences of the model we used the Bayesian approach and obtained 95% credible intervals for the model parameters [81].

As a second step we assessed the relationship of paternal care and paternity loss. For this we used a sub set of individuals for which both paternal care and paternity data were available. Paternal care, expressed as the proportional feeding rate of the male was used as the dependent variable. In addition, we used the absolute feeding rate in a separate model. Paternity loss (yes/no), brood number (1st/2nd), age and number of nestlings served as independent variables. We also included air temperature and song frequency in the model because they were related to paternal care in the previous model (see Results section). A generalized linear mixed model (glmer) with binomial distribution was used. Bird ID was included as a random effect to account for repeated measures. For the inferences of the model we used the Bayesian approach to obtain 95% credible intervals for the model parameters using an uninformed prior distribution, which is the equivalent of null hypothesis testing. Currently, the Bayesian approach is the only method that allows drawing exact inferences and avoids the difficulties of determining the degrees of freedom in mixed model analyses [81].

In addition, we assessed if male and female feeding rates were correlated. Using a linear mixed model, we contrasted these two parameters. Because both parent were always observed on the same days we included the identity of the couple as a random effect to account for repeated measures. We also did a Pearson’s correlation using the mean of all feeding observations.

As a third step, to assess which factors may have affected testosterone concentrations, the effect of paternity loss (yes/no), the GnRH treatment (before/after), the breeding stage during which the blood sample was taken (mating/feeding) and the brood sequence (first or second) on testosterone levels was tested using a linear mixed model (lmer). Bird ID was included as a random effect to account for repeated measures (because (a) all males were sampled twice to measure post capture and GnRH-induced testosterone concentrations, (b) 8 individual males were sampled during both breeding stages and (c) we included data from nestlings of first and second broods for 7 males).

Finally, we assessed which factors may have affected corticosterone concentrations. First, we tested whether corticosterone concentrations were related to the feeding proportion of males, the breeding stage (mating/feeding) and the brood sequence (first/second) using a linear mixed model (lmer). Bird ID was included as a random effect to account for repeated measures, because 3 males were sampled twice (during mating and feeding). In addition, separately we assessed if corticosterone was related to testosterone concentrations using a linear mixed model. Finally, in the subset of males for which data regarding paternity loss were available, we tested if corticosterone levels differed depending on paternity loss (yes/no) using a linear mixed model.

## Abbreviation

GnRH: Gonadotropin releasing hormone.

## Competing interests

The authors declare that they have no competing interests.

## Authors’ contributions

CPV and WG conceived the study and design the experimental set-up. CPV, WG and BA executed the experiments. CPV analyzed the data and wrote the first draft of the manuscript. CPV and WG wrote the final version of the manuscript. All authors read and approved the final manuscript.

## Supplementary Material

Additional file 1: Table S1 Characterization of 13 microsatellite loci for *Phoenicurus ochruros*. Primer sequences include information on fluorescence labels used and details of the multiplex PCR conditions per mix (temperature and cycles). C is the primer concentration in multiplex primer mix and NA is the number of alleles. Click here for file
