# Supplementary material for: Parental care, loss of paternity and circulating levels of testosterone and corticosterone in a socially monogamous song bird
Source: Front Zool. 2014 Feb 12;11:11. doi: 10.1186/1742-9994-11-11 (PMC3932846; doi:10.1186/1742-9994-11-11)
Supplement: Additional file 1: Table S1 — Characterization of 13 microsatellite loci for Phoenicurus ochruros. Primer sequences include information on fluorescence labels used and details of the multiplex PCR conditions per mix (temperature and cycles). C is the primer concentration in multiplex primer mix and NA is the number of alleles. [file 1742-9994-11-11-S1.pdf]

Additional file

Additional file 1: Table S1: **Characterization of 13 microsatellite loci for *Phoenicurus ochruros*.** Primer sequences include information on fluorescence labels used and details of the multiplex PCR conditions per mix (temperature and cycles). C is the primer concentration in multiplex primer mix and NA is the number of alleles.

Locus	EMBL/ GenBank Accession no.	Primer sequences (5' - 3')	C (µM)	Multiplex Mix	Size range (bp)	PCR T (°C)	PCR cycles	N_A
Asµ15-ZEST	AY172993.1	F: 6FAM-AATAGATTCAGGTGCTTTTTCC	0.6	A	100-160	57	24	16
		R: GGTTTTTGAGAAAATTATACTTTCAG	0.6					
DkiB102-ZEST	AY769673.1	F: PET-TTGCAACAGGAGGACAAGG	0.4	A	200-265	57	24	13
		R: CAGCAGCACTTCCCAATACA	0.4					
CcaTgu21	DV961016.1	F: VIC-GGCAGACATGATTGCATCC	0.4	A	189-253	57	24	8
		R: TCTCAGTGGTCATTGGAAAGTG	0.4					
TguEST09-021	CK313022.1	F: 6FAM-TGCTGAGGTATGATCTCTACCAAC	0.16	B	95-125	55	25	3
		R: GACCTACAGCCTGAACACACC	0.16					
TG02-088	DV579347.1	F: 6FAM-TGTGTGTTGACAGTATTCTCTTGC	0.4	B	250-310	55	25	14
		R: TTTAAACCTAATAAACGTCACACAGTC	0.4					
CcaTgu3	DV580602.1	F: VIC-CAAGTGCRGAAAGGAAAGTG	0.6	B	155-200	55	25	4
		R: TCAACTGCATCAGACTTCAAAA	0.6					
TguEST09-005	DV954446.1	F: PET-AACCCAACCAACAAAATTGG	0.24	B	146-186	55	25	6
		R: CCAACTATCAGTTTTACAAGGCATAC	0.24					
CcaTgu15	DV952837.1	F: NED-TTAATCCTAGGGTGTYGAGAGAAC	0.34	B	125-149	55	25	7
		R: CCTTTTTCCTTAAATTAKCTCAGCTT	0.34					
TG01-124	CK306631.1	F: 6FAM-AGTACTACTTGCCTGCAGAGTTTAT	0.4	C	390-440	55	25	5
		R: TGTGTATGGCAGCATTTACAA	0.4					
ADCYAP1	FJ464427.1	F: VIC-GATGTGAGTAACCAGCCACT	0.22	C	150-185	55	25	11
		R: ATAACACAGGAGCGGTGA	0.22					
Tgu7	DV948303.1	F: PET-CTTCCTGCTATAAGGCACAGG	0.46	C	89-135	55	25	9
		R: AAGTGATCACATTTATTTGAATAT	0.46					
Mcyµ4	U82388	F: PET-ATAAGATGACTAAGGTCTCTGGTG	1.3	D	115-175	52	24	10
		R: TAGCAATTGTCTATCATGGTTTG	1.3					
Gf06	AF081930.1	F: NED-GCTATTGAGCTAACTAAATAAACAACT	0.6	D	120-195	52	24	11
		R: CACAAATAGTAATTAAAAGGAAGTACC	0.6					
